# Old and New Biological Therapies for Psoriasis

**DOI:** 10.3390/ijms18112297

**Published:** 2017-11-01

**Authors:** Kirsten Rønholt, Lars Iversen

**Affiliations:** Department of Dermatology, Aarhus University Hospital, 8000 Aarhus, Denmark; lars.iversen@clin.au.dk

**Keywords:** psoriasis, psoriasis arthritis, biological therapy

## Abstract

Biological therapy became available for psoriasis with the introduction of alefacept at the beginning of this century. Up to then, systemic treatment options comprised small molecule drugs, targeting the immune system in a non-specific manner. The first biologics targeted T-cell activation and migration and served as an alternative to small molecules. However, significant improvement in outcome was first accomplished with the introduction of tumor necrosis factor-α inhibitors that were already approved for other inflammatory disorders, including rheumatic diseases. Along with the progress in understanding psoriasis pathogenesis, highly targeted and effective therapies have since developed with the perspective not only to improve but to clear psoriasis. These accomplishments enable future achievement of advanced goals to individualize treatment best suited for each patient. Mechanistic studies with patients treated with the new highly targeted biologics may guide us towards these goals. This review offers an overview of biologics developed for psoriasis and illustrate a historical progress in the treatment of this common chronic inflammatory skin condition.

## 1. Introduction

Psoriasis is present worldwide, but with varying prevalence (e.g., 0.24% in Taiwan vs. 8.5% in certain areas of Norway) [1,2]. Traditional co-morbidities associated with psoriasis is psoriasis arthritis (PsA) (prevalence of 10–30% [3,4]), inflammatory bowel disease [5], and cancers (especially cancers associated with alcohol drinking and smoking) [6]. However, recent studies have established solid evidence for psoriasis being associated with a palate of lifestyle co-morbidities, the metabolic syndrome and the derived consequences diabetes and cardiovascular disease [7,8,9,10,11]. These factors are more strongly associated with patients suffering from severe psoriasis compared with mild psoriasis [10]. Cardiovascular disease involves T-cell signaling pathways common to the inflammatory process of psoriasis. A state of low-grade inflammation may be a contributing factor to the reduced life expectancy in psoriasis patients [9,12,13]. With this knowledge, the rationale of effectively and systemically treating inflammation in patients with moderate to severe psoriasis becomes even more profound.

Small molecules were the first breakthrough in systemically treating psoriasis. They mainly targeted the immune system in a non-specific manner. Psoriasis area severity index (PASI) 75, defined as a 75% reduction in PASI, has for decades been the gold standard for primary outcome in randomized controlled trails of therapies for psoriasis. The PASI75 response rate for methotrexate, a conventional first-choice small molecule drug for systemic treatment of psoriasis, is typically 35.5–41% [14,15].

Today we are standing in the middle of the second breakthrough of systemic treatments for psoriasis with the development of biologics. With these highly targeted and effective therapies, PASI90 and PASI100 have become the new, realistic gold standard endpoint measurements in clinical trials [16].

Biologics developed for psoriasis are for the indication of patients with moderate to severe psoriatic disease that are candidates for systemic treatment or phototherapy. Some of the biologics have also received approval for PsA. Most results on the latest biologics developed for psoriasis are based on randomized studies with placebo as the control arm, whereas only a few head-to-head studies have been completed. Also, there is a need for data on long-term efficacy and safety that cannot be generated from the relative short-term clinical trials required for drug approval. Therefore, it is not possible, at this point, to draw conclusions on the most favorable drug within the group of the newest, highly targeted biologics. One approach is indeed to identify the best drug over others on average. However, another future approach may be to focus research on the link between psoriasis, genetics and co-morbidities in context with the mechanism of new biologics. This valuable knowledge may generate the possibility of an individualized treatment according to a mapped, personal “psoriasis-profile” for each patient.

## 2. Definition of Biological Therapy

Psoriasis has for many years been considered a skin disease that originated primarily from abnormal behavior of keratinocytes. During the last 30 years, more substantial evidence has been provided that the immune system plays a crucial role in both initiating and maintaining psoriasis. In 1979, Mueller and Hermann conducted a pilot study to investigate the effect of ciclosporin on rheumatoid arthritis and included four patients with PsA as well [17]. To their surprise, ciclosporin had excellent effect on psoriasis skin lesions. The authors discussed ciclosporins anti-lymphocytic effect, mainly on T-cells, as part of the mechanism. Years later, it was demonstrated that in mice with severe combined immunodeficiency, injected activated autologous peripheral blood mononuclear cells obtained from psoriasis patients, converted transplanted non-lesional human psoriasis skin into psoriatic plaques [18]. These results strongly supported the theory of immune cells as main actors in driving psoriasis.

Ciclosporin is a small molecule drug, and more small molecules were introduced in the treatment of psoriasis where topical agents were insufficient: hydroxyurea, retinoids, fumaric acid esters, and methotrexate. Apremilast is a phosphodiesterase-4 inhibitor that was more recently marketed for psoriasis, and a new formulation of dimethyl fumarate was marketed in Europe in June 2017. More are in clinical development, including Piclidenoson/CF101 (adenosine A3 receptor inhibitor) and BMS-986165 (tyrosine kinase 2 inhibitor) (www.clinicaltrial.gov accessed on June 2017: NCT03168256 and NCT02931838).

The terms biological therapy/biological product/biopharmaceutical/biological/biologic are used haphazardly, but actually comprise a variety of products with natural origin, e.g., vaccines, blood and blood components, gene therapy, and recombinant protein sources [19]. However, today we most often aim to refer to the subgroup of large, complex molecules that represent targeted therapy, including monoclonal antibodies and receptor fusion proteins. In this review, we will in the following use the term “biologic”.

Compared to small molecules, biologics are large molecular-weight proteins that need to be injected, since they would become degraded in the gastro-intestinal tract if orally administered. Small molecules have a small molecular weight (<1000 Da), are administered orally or topically and their target is less specific compared with biologics [20]. Whereas small molecular drugs are able to cross the cell membrane, biologics act outside or on the surface of cells. Furthermore, biologics require engineering from live, specialized cells, whereas small molecules are simpler and chemically synthetized [21].

The nomenclature for receptor fusion proteins and monoclonal antibodies follow the rules set by International Nonproprietary Names selected by the World Health Organization. “-Cept” is used as the stem to identify the drug as a receptor molecule, and “-mab” is used as the stem for monoclonal antibodies. Antibody origin is indicated by introducing a “-u-” for fully human origin (e.g., secukinumab). Antibodies that origin from both human and mouse are humanized, “-zu-” (e.g., ixekizumab or chimeric, “-xi-” (e.g., infliximab) [22].

## 3. Psoriasis Pathogenesis

Biologics are the proof-of-concept for the significant progress that has been achieved in understanding the complex pathogenesis of psoriasis. The specific targets of biologics have out-pointed crucial cytokines, including tumor-necrosis-factor (TNF)-α, interleukin (IL)-23 and IL-17, for the development and maintenance of the skin changes in psoriasis. The sequences of cellular events involved in IL-23 and Th17 signaling are referred to as the IL-23/Th17 signaling pathway, and is one of the most studied in psoriasis [23]. In order to give a better understanding of where in the immune system the various biologics are targeting, an overview is given in the following, of the most important cells and cytokines involved in development of psoriasis.

Psoriasis pathogenesis involves both the innate—and the adaptive immune response. Though, psoriasis is mainly considered a T-cell mediated disease, and the T-cell targeting biologics, alefacept and efalizumab substantiated the role of T-cells as primary modulators. The IL-23/Th17 pathway not only involves different subgroups of T-cells, but also include, e.g., dendritic cells, macrophages, neutrophils, and keratinocytes. The initiation of this inappropriate immune response is still being elucidated, but the combination of a predisposing genotype and external factors as skin trauma, known pharmaceuticals, bacterial and viral infections, and stress have been suggested as key triggers.

Traditionally, pathogenesis of psoriasis is considered in two phases, the initiation phase and the maintenance phase. Some of the triggers of psoriasis are known to disrupt or stress the keratinocytes in the epidermis which in turn release self-deoxyribonucleic acid (DNA) and the anti-microbial peptide LL-37 (cathelicidin). Besides self-DNA, pathogen-derived DNA is able to form a complex with LL-37. The complex binds to toll-like receptor 9 on plasmacytoid dendritic cells in the dermis. The plasmacytoid dendritic cell secretes type 1 interferons (IFN-α and -β), TNF-α, IL-6, and IL-1β that stimulate the local myeloid dendritic cells to migrate to the local draining lymph nodes. Upon contact with the resting naive T-cells, they secrete cytokines including TNF-α, IL-12 and IL-23, which induce the T-cell to differentiate into mature Th1-, Th17-, and Th22-cells. When returning to the skin, these specific T-cell linages release TNF-α, IFN-γ, IL-17A and -F, and IL-22 that stimulate keratinocytes to proliferate and leads to an altered differentiation. Erythema due to angiogenesis, dilated and tortious vessels in the dermal papilla, thickening of the skin due to acanthosis, and scaling due to an accelerated proliferation and altered differentiation of keratinocytes develop. Around 10 years ago, the IL-17 producing subtype of T-cells, Th17-cells, was identified [24]. IL-17A has though been found not only to be linked to the cluster of differentiation (CD)4 + T-cells. CD8 + T-cells, γδ T-cells, natural killer cells, mast cells and neutrophils are all sources of IL-17A [25,26,27]. Furthermore, keratinocytes are a source of IL-17C [28].

Upon receptor stimulation of keratinocytes, they themselves produce cytokines, e.g., TNF-α, IL-6, IL-1β, and chemokines, chemokine (C–X–C motif) ligand (CXCL) 8, CXCL10, and chemokine (C–C motif) ligand 20 (CCL20). The pro-inflammatory cytokines enhance the activation of the present mature T-cells and close the loop of a chronic inflammation cycle that represent the maintenance phase. The keratinocyte secreted chemokines recruit neutrophils from the circulation that enter the skin and collect into the characteristic microabcesses of Munro in the epidermis. Macrophages are also recruited to the site of inflammation and produce TNF-α [26,29]. Once established, this pathological cycle of inflammation can be activated at distant locations with or without the initial trigger factors.

**Figure 1:**
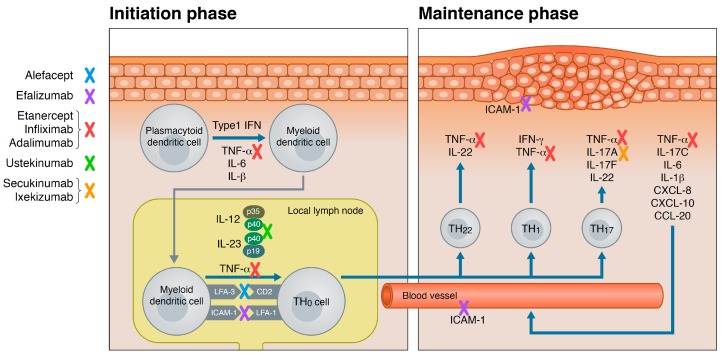
The targets for old and new biologics within the immunological process of psoriasis are illustrated. Included are biologics that have been approved by the United States Food and Drug Administration (as of June 2017). IFN: Interferon. TNF: Tumor necrosis factor. IL: Interleukin. LFA: Lymphocyte function-associated antigen. CD: Cluster of differentiation. ICAM: Intercellular adhesion molecule. CXCL: Chemokine (C–X–C motif) ligand. CCL: Chemokine (C–C motif) ligand.

In Figure 1, the site of action of biologics that has been marketed for the treatment of psoriasis is illustrated. 

## 4. Biologics for Psoriasis

No definition on biologics for psoriasis categorizes them in old and new therapies. However, the oldest group of biologics encompasses molecules that target the activation and migration of T-cells, alefacept and efalizumab. Therefore, biologics targeting TNF-α were developed, and are often referred to as first-generation biologics: etanercept, infliximab, and adalimumab. Second-generation biologics emerged from 2009 with antibodies targeting the IL-23/Th17-pathway (Table 1). Ustekinumab, secukinumab, ixekizumab, brodalumab, and guselkumab have received United States Food and Drug Administration (FDA) approval, and more are in late, clinical development for psoriasis: tildrakizumab and risankizumab.

In this review, PASI75 will be reported from clinical studies as measurement of efficacy. This endpoint has been the primary endpoint for the majority of studies and illustrates the development in efficacy from the first biologics up till today. Calculation of PASI encompasses the physician’s assessment of the degree of the lesions’ redness, thickness, scaliness, and the proportion of area involved. For years, PASI75 has been the gold standard endpoint, but today PASI90 and PASI100 are being used more extensively, as therapies have become more efficient and patient expectations to treatment higher. Moreover, there is supporting evidence that further improvement in patient quality of life is achieved with a PASI90—and PASI100 response, compared with a PASI75 response [30,31]. Other often-reported endpoints include Physician Global Assessment (PGA)/Investigator’s Global Assessments and calculation of the affected body surface area (BSA). The difficult-to-treat sites, scalp and nails, are assessed by Psoriasis Scalp Severity Index and Nails Psoriasis Severity Index. A tool to measure quality of life in patients with dermatological conditions is Dermatology Life Quality Index, and furthermore short-form-36 has been developed for patients with psoriasis specifically. They are important tools to confirm that the improvements documented with the calculation of objective measurement tools are also clinical meaningful to the patient. More patient-reported outcome measurements specific for psoriasis are available and being used in newer clinical trials to document efficacy of the medication being tested on function and psychosocial aspects which are factors greatly affected by psoriasis.

### 4.1. T-Cell Targeted Biologics

#### 4.1.1. Alefacept

Alefacept was the first biologic approved by the FDA for treatment of psoriasis [32,33]. Alefacept is a human lymphocyte function-associated antigen (LFA)-3/immunoglobulin (Ig) 1 fusion protein. It binds to CD2 molecules on the surface of activated T-cells, and thereby blocks co-stimulation of T-cells by the antigen presenting cell (APC). In addition, it selectively targets memory-effector T-cells, thereby both acts to inhibit activation of T-cells and deplete memory T-cells [33]. Due to alefacept’s mechanism of action, the drug was expected to provide a relatively long-term remission even without treatment [34]. Two phase III studies reported results on alefacept as intra-muscular (I.M.) injection and as intra-venous (I.V.) administration, respectively, demonstrating similar but only modest efficacy. Only I.M. administration of alefacept was approved for treatment of psoriasis. In the trial program, alefacept I.M. was administered as 15 mg once weekly for 12 weeks and I.V. administration as 7.5 mg once weekly for 12 weeks. The authors both included PASI75 response rate two weeks after the last dose of alefacept, and an overall response rate after 12 weeks of treatment and 12 weeks of follow-up as more patients responded to treatment at a later time point. The proportion of patients achieving PASI75 at week 2 post-treatment was 21% (I.M. alefacept) and 14% (I.V. alefacept) compared with placebo 5% (I.M. alefacept) and 4% (I.V. alefacept). The overall PASI75 response rate was 33% (I.M. alefacept) and 28% (I.V. alefacept) compared with placebo as high as 13% (I.M. alefacept) and 8% (I.V. alefacept) [35,36]. After a 12-week treatment-free period, a second course of I.V. alefacept improved the overall PASI75 response to 40% [35]. Median duration of remission (time to retreatment or maintenance of PASI50) was 7 to 10 months for both I.M. and I.V. administration in phase II and III studies [34]. Although efficacy of alefacept was only modest, the drug was an option to patients not controlled on conventional systemic treatment. The authors commented that at this time point (in 2002) complete clearing was hoped for but not expected in psoriasis [35,37]. 

As more biologics emerged, alefacept became inferior in treatment of psoriasis. A study from 2013 on cost-effectiveness of biologics for psoriasis showed overall that alefacept was the least cost-effective biologic agent compared with adalimumab, etanercept, infliximab, and ustekinumab [38]. In 2011 alefacept was withdrawn from the market.

#### 4.1.2. Efalizumab

Efalizumab was approved by the FDA the same year as alefacept, and was the first biologic approved in both the United States and Europe. The drug is a humanized monoclonal IgG1 antibody, directed against CD11a, the α-subunit of LFA-1. It leads to blockade of the interaction between LFA-1 and Intercellular Adhesion Molecule 1 (ICAM-1), which similar to alefacept interferes with the co-stimulation of T-cells when contacted by the APC. As ICAM-1 is expressed in a variety of tissues, the sites of action are plural for efalizumab compared with alefacept. Efalizumab affects extravasation of circulating lymphocytes and the interaction of activated T-cells with keratinocytes [39,40].

In two phase III studies, efalizumab 1.0 mg/kg once weekly for 12 weeks (initial conditioning dose of 0.7 mg/kg) demonstrated PASI75 response rates of 26.6% and 31.4% versus 4.3% and 4.2% in the placebo group [41,42]. Continuous treatment through 24 weeks improved PASI75 to include 43.8% of the treatment group [42]. A third phase III open-label trial demonstrated a higher PASI75 rate at week 12 (41.3%) with the double dose efalizumab (2 mg/kg once weekly). Patients who had achieved ≥PASI50 were included in a second phase of efalizumab 1 mg/kg once weekly for up to 36 months of therapy. Intention to treat analysis (last observation carried forward) showed that at week 36 the proportion of patients with a PASI75 response was stable on 45.4% and, as expected, higher in an additional as-treated analysis [43].

Although not obvious from clinical trials, the extended mode of action of efalizumab compared with alefacept was reflected in an improved efficacy for treatment of psoriasis in a meta-analysis. Efficacy of alefacept, efalizumab, etanercept, and infliximab was ranked, resulting in lowest rank for alefacept and efalizumab, although efalizumab with superior rank compared to alefacept [32].

Problems with safety became clear in the post-marketing phase. Association between long-term treatment with efalizumab and development of the rare but life-threatening disorder progressive multifocal leukoencephalopathy (PCL), was established [44]. PCL is a rapidly progressive infection of the central nervous system caused by JC-virus. Therefore, efalizumab was voluntarily withdrawn from the market in 2009 [45].

### 4.2. Tumor-Necrosis-Factor (TNF)-α Inhibitors

#### 4.2.1. Etanercept

Already approved for the treatment of PsA, etanercept was the first TNF-α inhibitor FDA approved for treatment of psoriasis in 2004 [46,47]. In 2009, the European Commission extended the indication for adults to children from the age of six years. First in 2016, FDA also approved etanercept for the use in children down to the age of four years [48].

Etanercept is a recombinant human TNF-receptor fusion protein. It consists of two extracellular domains of human soluble TNF receptor units that bind TNF-α, both soluble and membrane-bound, and a Fc-fragment of human IgG that stabilizes the molecule. As a dimeric molecule, it can bind two TNF-α molecules and act as a competitive inhibitor of endogenous TNF-α [49]. TNF-α is produced by e.g., dendritic cells, Th1-, Th22-, and Th17 cells, macrophages, and keratinocytes with multiple targets in psoriasis pathogenesis. TNF-α inhibitors are therefore considered targeted therapy, but with a widespread target, compared to second-generation biologics.

Phase III results reported that etanercept 50 mg twice weekly resulted in achievement of PASI75 at week 12 in 47–49% of patients compared with placebo (3–5%) [49,50,51]. Further treatment with etanercept 50 mg twice weekly improved PASI75 response rate; at week 24, the PASI75 response was 59% (last observation carried forward) [50]. Similar results were achieved in an open label extension of a phase III study up to week 96 [52]. Label dosing for etanercept is however, 50 mg every other week after the initial 12-week induction treatment. 

Etanercept has been used as the counterpart in several head-to-head studies both within first-generation biologics (described in the last section of Section 4.2) as well as newly approved second-generation biologics (described under the respective second-generation biologics). Recently, etanercept has been compared with tofacitinib—that is biologic versus small molecule therapy—with similar results on PASI75 [53]. Tofacitinib is though not yet approved for the indication psoriasis.

#### 4.2.2. Infliximab

In 2006, the FDA approved the use of infliximab for psoriasis, one year after the approval for treatment of PsA. Infliximab is a chimeric IgG1 monoclonal antibody (human antibody constant regions and murine variable regions) that binds to and neutralizes biological activity of TNF-α by binding soluble and membrane-bound TNF-α [54].

In terms of efficacy, infliximab demonstrates favorable performance; in a meta-analysis of TNF-α inhibitors and ustekinumab, infliximab and ustekinumab were the most effective treatments with similar performance [55]. Two phase III studies reported that infliximab 5 mg/kg at week 0, 2, and 6 resulted in PASI75 responses at week 10 of 75.5% and 80% compared with 1.9% and 3.0% with placebo [56,57]. Similar results on efficacy were found in a randomized, controlled trial of Chinese patients with moderate to severe psoriasis [58]. Of all the above studies, the lowest proportion of patients achieving PASI75 at week 10 was in a population of Japanese patients with moderate to severe psoriasis (PASI75; infliximab = 68.6% versus placebo = 0%) [59].

The chimeric composition of infliximab may render the drug more immunogenic compared with etanercept and adalimumab. In theory, this may lead to increased production of neutralizing anti-drug-antibodies (ADA) and the risk of infusion reactions. Infusion reaction, has been seen in 3–23% of psoriasis patients treated with infliximab and was in severe cases the direct cause for discontinuation of treatment [56,57,60,61]. Immediate infusion reactions include e.g., pruritus, flushing, hypertension, headache, and rash, whereas late infusion reactions are less frequent and is of the serum sickness type [62]. Intermitted treatment with infliximab as needed has been studied with the intention to improve outcome, but resulted in increased risk of infusion reactions from 6.2–7.2% (normal regime) to 9.2–11.1% (as-needed) of all infusions [56]. Infliximab in combination with methotrexate has not been approved for psoriasis as it is for e.g., rheumatoid arthritis. The combination with methotrexate, decreases the risk of loss of effect. Moreover, infliximab as monotherapy is more often associated with infusion reactions [60,63]. 

#### 4.2.3. Adalimumab

Adalimumab was approved by the FDA in 2005 for the treatment of PsA and three years later received approval for treatment of psoriasis [64]. So far, adalimumab has received licenses in only Europe (in 2015) for the treatment of children from four years of age. Adalimumab is a fully human monoclonal antibody of the IgG1 isotype that binds soluble and membrane-bound TNF-α like infliximab [65].

Results from a phase III study showed, that with adalimumab 80 mg week 0 followed by 40 mg at week 1 and then every other week, 71% achieved a PASI75 response at week 16 compared with 7% in the placebo group [65]. An extension of the phase III study (open label), showed that 76% of patients with at least a PASI75 response at week 16 and 33 (sustained responders), maintained PASI75 after 160 weeks of continuous treatment (measured as last observation carried forward) [66].

Results on efficacy were similar in Japanese and Chinese patients with moderate to severe psoriasis [67,68].

Two additional phase III studies have been conducted. One was a head-to-head study comparing adalimumab with increasing doses of oral methotrexate, and showed that at week 16, 79.6% of adalimumab-treated patients achieved PASI75, compared with 35.5% for methotrexate and 18.9% for placebo. In addition, adalimumab’s onset of action was more rapid compared with methotrexate [14]. There are however, some concerns that need to be addressed. The relatively high effect in the placebo group raises concern about the validity of the results. Furthermore, in this study, the starting dose of methotrexate was 7.5 mg/kg/week. In daily clinic, starting doses of oral methotrexate are more often 12.5–15 mg/kg/week [69,70]. Moreover, results from a recent randomized study on methotrexate as injection therapy, indicate that injection therapy is more effective and has a faster onset of action when considering previously reported results on oral methotrexate [15]. A randomized study on oral versus injection methotrexate is though needed to draw conclusion.

The third phase III study examined combination therapy with adalimumab + calcipotriol/betamethasone and adalimumab + vehicle. At week 16, the PASI75 response was 64.8% for the combination versus 70.9% for adalimumab as monotherapy with no significant difference. However, combination therapy resulted in a more rapid response within the first four weeks of treatment [71]. Altogether, the clinical studies above have demonstrated a PASI75 response at week 16 for adalimumab to comprise around 70% of patients being treated.

The group of TNF-α inhibitors share the same target. However, due to structural differences of the molecule for each drug, the overall mechanism of action differs, which is reflected in terms of efficacy and adverse events. PASI75 responses at week 10, 12 or 16 reported in the phase III studies above for the group of TNF-α inhibitors, range from 47% (etanercept) up to 80% (infliximab), with results on adalimumab close to infliximab. Studies specifically designed to compare the three TNF-α inhibitors have brought more supportive data for infliximab to be the most effective and etanercept the least effective. A head-to-head study and a meta-analysis, have found infliximab superior in terms of PASI compared to etanercept, and the meta-analysis categorized etanercept as the least effective compared with infliximab and adalimumab [72,73].

For each of the TNF-α inhibitors, there is a risk of developing ADA. The clinical relevance of ADA on loss of efficacy and adverse events during treatment is not always clear. Enzyme-linked immunosorbent assay have been used for detection of ADA which may have contributed to the large variation reported in different studies in addition to the fact that some patient samples are tested inconclusive to whether or not they are positive for ADA [74]. Patients positive for ADA have also been seen to test negative at a later time point with continues treatment [51]. Moreover, a dose-response relationship for the development of ADA has not clearly been demonstrated [56,57]. Some clinical trials determine the status of ADA as being neutralizing or non-neutralizing, but there is no consensus opinion on the consequence of this distinction [49,50,51]. In spite of these challenges, there has been some evidence that the presence of ADA in patients treated with infliximab and adalimumab experience decreased clinical response (and is associated with reduced plasma concentration of TNF-α inhibitor), whereas ADA against etanercept has no effect on clinical response [74,75]. Detection of declining plasma TNF-α concentration may be a better tool to detect clinical influence of developed ADA.

For the group of TNF-α inhibitors, rare but severe adverse events have been reported and included in the drug label. In the nature of being rare, cases most often did not reach significance compared to placebo or control treatment in clinical trials of psoriasis. Severe adverse events include serious infections (sepsis and opportunistic infections such as tuberculosis (TB)), malignancies (e.g., lymphoma and non-melanoma skin cancers), multiple sclerosis, lupus, and congestive heart failure. The adverse events most frequently reported for etanercept and adalimumab are injection site reactions, upper respiratory tract infections, sinusitis, and headache. Infusion-related reactions and abdominal pain are additional frequent adverse events with infliximab treatment, besides upper respiratory tract infections, sinusitis, pharyngitis and headache [76,77,78].

More mechanistic properties have been described for TNF-α inhibitors to enhance development of an active infection with Mycobacterium Tuberculosis. The relatively widespread alterations within both the innate and adaptive immune system as well as disruption of the TB granulomas upon treatment with TNF-α inhibitors have been proposed to increase the risk of active TB [79]. Data on TNF-inhibitors and the risk of TB, is mainly based on patients with rheumatic or inflammatory bowel disease which differ from psoriasis patients in the aspect of disease, co-morbidities, and concomitant treatments. However, the same conclusion has been found in a small group of patients treated for psoriasis where 1.08% developed TB despite of prophylactic screening before initiation of TNF-α inhibitor [80].

Although no long-term data has been published on adverse events for infliximab, an unpublished meta-analysis conducted by a specialist committee on behalf of The Danish Council for the use of Expensive Hospital Medicines indicates a larger risk of severe adverse events for infliximab compared with etanercept and adalimumab [81]. Conclusively, although infliximab seems superior in terms of efficacy, a higher risk of serious adverse events and the risk of infusion reaction which spans from mild to severe must be taken into account in the decision making of systemic therapy.

### 4.3. IL-12/IL-23 Inhibitor

#### Ustekinumab

Psoriasis was the first inflammatory disease for which ustekinumab was licensed by the FDA. This is unlike the TNF-α inhibitors that were already approved for Crohn’s disease and/or a range of rheumatic diseases (including PsA) when receiving FDA approval for psoriasis. Four years later, in 2013, ustekinumab was also approved for PsA, and in 2016 for Crohn’s disease.

Ustekinumab is a human IgG1 monoclonal antibody that targets the shared protein subunit p40 of IL-12 and IL-23 [82]. Thereby it inhibits the action of these two cytokines, secreted by the myeloid dendritic cells upon activation and differentiation of naive T-cells into Th1- and Th17-cells. Different from the former biologics registered for treatment of psoriasis, ustekinumab specifically targets the IL-12/Th1—and IL-23/Th17 pathways important in psoriasis pathogenesis. With this novel approach of interfering with the immune system, ustekinumab paved the way for a new strategy in drug development for psoriasis.

Two phase III studies on patients with psoriasis demonstrated that treatment with ustekinumab 90 mg on week 0 and 4 resulted in PASI75 responses at week 12 of 66.4% and 75.7%, respectively, compared with 3.1% and 3.7% in the placebo group [82,83]. With continuous treatment, 90 mg every 12 weeks, similar PASI75 responses were reached in the two studies, 78.5% and 78.6%, at week 28. In general, PASI75 responses were lower in patients randomized to receive ustekinumab at the dose of 45 mg. In three Asian phase III studies including Taiwanese/Korean, Chinese, and Japanese patients, respectively, ustekinumab 45 mg resulted in PASI75 responses in the range of 59.4–82.5%, compared with placebo 5–11.1% [84,85,86]. These results reflect the labeling dosage of ustekinumab: For patients with a bodyweight ≤100 kg the dose of ustekinumab is 45 mg and with a body weight of >100 kg the dose of ustekinumab is 90 mg.

In subsequent studies based on data from registries representing real-life data it seems as ustekinumab, compared with the three anti-TNF-α agents, has a significantly longer drug survival [87,88,89]. In the short term (median 16 weeks), prior treatment with a TNF-α inhibitor did not influence efficacy of ustekinumab, whereas number of prior biologics affected the long-term outcome [87,90]. In two phase III randomized studies, 68.7% and 70% of the patients completed 5 years of ustekinumab treatment with 72% and 78.6% having PASI75 response on ustekinumab 90 mg every 12 weeks (dosing flexibility permitted) [91,92]. By only including initial responders, PASI75 response was 80.8% (78.8% when measured as last observation carried forward) [91]. This correlated with a retrospective study on patients with psoriasis treated with a TNF-α inhibitor or ustekinumab, that demonstrated longer drug survival and the lowest proportion of loss of efficacy for ustekinumab compared with the TNF-α inhibitors. For all patients (biologically naive and non-naive) 70% remained on ustekinumab after 4 years of treatment [87].

One single head-to-head study comparing the effect of ustekinumab with etanercept has been published and supports, according to the label, the above-mentioned results on efficacy; at week 12, efficacy of ustekinumab at a dose of 45 or 90 mg was superior to etanercept [93].

Follow-up from 3 to 5 years indicate a possible safer profile of ustekinumab compared with the TNF-α inhibitors with no increased risk of serious infections and malignancies compared with placebo [87,94]. However, serious adverse events included in the label of ustekinumab are infections (e.g., TB) and malignancies (mainly non-melanoma skin cancers) [95]. The risk of TB is, however, mainly based on the fact that genetic deficiency of IL-12/IL-23 is associated with increased risk of disseminated infections from e.g., mycobacteria [96]. TB has only been reported in one case report and one clinical trial of Taiwanese patients receiving ustekinumab for psoriasis [85,97]. The most frequent adverse events for ustekinumab are nasopharyngitis, upper respiratory tract infection, headache, and fatigue [95].

### 4.4. IL-17 Inhibitors

#### 4.4.1. Secukinumab

Secukinumab was the first IL-17A inhibitor approved in 2015 for treatment of patients with psoriasis, and about one year later secukinumab was also approved for PsA. It is a fully human anti-IL-17A IgG1 monoclonal antibody that selectively binds and neutralizes IL-17A. The IL-17 cytokine family comprises six members, IL-17A-F. Both IL-17A and IL-17F are secreted by Th17-cells and other immune cells, as described previously. Interleukin-17A is about 10–30-fold more potent than IL-17F, whereas the IL-17A/IL-17F heterodimer has intermediate activity [98].

In phase III studies, the proportion of patients who achieved PASI75 at week 12 was 75.9–86.7% with secukinumab 300 mg (administered once weekly for 4 weeks starting at week 0, then every 4 weeks) and 0–4.9% with placebo [99,100,101]. A post-hoc analysis including Japanese patients only, showed similar responses [99]. Head-to-head studies demonstrated secukinumab to be superior compared with both etanercept and ustekinumab [99,102,103]. Although the difference in PASI75 response was only small for the comparison of secukinumab to ustekinumab. Contrary, the difference was more pronounced when measuring the proportion of patients achieving a ≥PASI90 response [102,103]. After 52 weeks of treatment, the proportions of patients (secukinumab versus ustekinumab) with a PASI90 response were 76% versus 61% and the proportions with a PASI100 response were 46% versus 36% [102]. Already at week 1, PASI75 response rates were significantly higher for secukinumab compared with ustekinumab, and at week 4 the proportion of patients achieving PASI75 with secukinumab was 50.0% compared with 20.6% with ustekinumab [102,103]. 

Conclusively, compared with older biologics, secukinumab has a faster onset of action. The drug is highly effective, and compared with ustekinumab, secukinumab has higher PASI90—and PASI100 response rates.

Pooled safety data from 10 phase II and phase III studies have shown that secukinumab was comparable to etanercept over 1 year. An exception was a higher rate of uncomplicated mucocutaneus candida infections in patients treated with secukinumab compared with etanercept [104].

#### 4.4.2. Ixekizumab

Ixekizumab is a humanized IgG4 anti-IL-17A monoclonal antibody, which like secukinumab neutralizes IL-17A. FDA approved ixekizumab for treatment of psoriasis in 2016 [105]. Ixekizumab is not yet approved for PsA, but is undergoing clinical development [106,107].

Results from two independent phase III studies in psoriasis were reported in the same publication, and showed similar results. PASI75 responses reported for the treatment regimen of ixekizumab 160 mg as starting dose followed by 80 mg every 2 weeks were 87.3% and 89.7% at week 12 (for some patients, effect was seen as early as week 1), and was significantly higher than both placebo (7.3% and 2.4%) and treatment with etanercept (53.4% and 41.6%). Results on PASI90 and PASI100 were also significantly higher compared with placebo and etanercept ((PASI90: ixekizumab = 68.1% and 70.7%; placebo = 3.1% and 0.6%; etanercept = 25.7% and 18.7%), (PASI 100: ixekizumab = 37.1% and 40.5%; placebo = 0% and 0.6%; etanercept = 7.3% and 5.3%)) [30]. Combined long-term data from the two phase III studies (UNCOVER 2 and 3) as well as a third phase III study (UNCOVER 1) were published in 2016, and showed in general that >70% maintained treatment responses (PASI75 responses for UNCOVER 1 and 2 and PASI90 responses for UNCOVER 3) up to week 60 with ixekizumab 80 mg every 4 weeks as extension dose [108]. In Japanese patients with psoriasis results from a phase III study on ixekizumab have been even better. However, the study was only based on 78 patients with moderate to severe psoriasis, and designed as an open-label study with no placebo or control group. After 12 weeks of treatment almost all patients achieved PASI75 (98.7%) and 83.3% achieved PASI90 [109]. Results from an extension of this study showed that at week 52, 92.3% had a PASI75 response and 80.8% had a PASI90 response [110].

More sub-analyses that underline the effectiveness and usability of ixekizumab have been conducted on the basis of the former phase III trials and published separately: High treatment response has been demonstrated after switching to ixekizumab in etanercept non-responders [111]. The difficult to treat psoriasis areas, hands and feet, were improved by treatment with ixekizumab [112]. Treatment with ixekizumab has also been shown to improve patient-reported work productivity, measured by the Work Productivity and Activity Impairment Psoriasis score [113].

Safety data is available from seven phase I-III studies of patients treated with ixekizumab (12-week induction period and 60 weeks of maintenance). In the first 12-week period comparison with etanercept was eligible and showed similar safety profile, but with a higher frequency of uncomplicated mucocutaneus candida infections. Also, the exposure-adjusted incidence rate of injection site pain was higher in patients treated with ixekizumab every two weeks compared with etanercept. The maintenance period showed no unexpected safety issues [114]. 

#### 4.4.3. Brodalumab

The IL-17 receptor family comprises five receptor subunits IL-17RA–IL-17RE. Brodalumab is a fully human anti-IL-17RA IgG2 monoclonal antibody and thereby blocks IL-17 family members that act via IL-17RA, including IL-17A, IL-17A/F, IL-17F, IL17-C, and IL-17E (IL-25) [115,116]. Like IL-17A, IL-17F has been ascribed a pro-inflammatory role in psoriasis, e.g., through induction of IL-6 and IL-8 in keratinocytes and regulation of CCL20 and human beta-defensin-2 [117,118,119]. Expression of IL-17C, but not IL-25, is increased in psoriatic lesions, which indicates a less important role for IL-25 in psoriasis [116,120].

Compared with secukinumab and ixekizumab that target IL-17A alone, the ability of brodalumab to block the effects of more IL-17 cytokines involved in psoriasis by interacting with IL-17RA may contribute to higher efficacy. However, no head-to-head studies have been conducted for the comparisons of brodalumab against secukinumab or ixekizumab.

Three phase III studies have been completed (AMAGINE 1–3), where results from AMAGINE 2 and 3 were published together. At week 12, the proportion of patients achieving a PASI75 response with brodalumab 210 mg every other week (2 doses on week 1) was very similar among the three phase III trials, 83–86%, versus placebo 3–8% [121,122]. Efficacy compared with ustekinumab was measured at the level of a PASI100 achievement at week 12, and was significantly in favor of brodalumab (PASI100: brodalumab, AMAGINE 2 and 3: 44% and 37%. Ustekinumab, AMAGINE 2 and 3: 22% and 19%). Patients receiving brodalumab were re-randomized after the initial 12 weeks to four different dosing regimens (patients were stratified on i.a. response at week 12), where inadequate responders received rescue therapy from week 16 with brodalumab 210 mg every other week. In AMAGINE 2 and 3, 90% and 91% respectively, remained in the study at week 52. Treatment with the highest dose of brodalumab, 210 mg every other week, from week 12 to 52 (including placebo switched to treatment), >60% had a static PGA score of 0 or 1 (clear or almost clear skin) at week 54 (missing data and patients with inadequate response were imputed as non-responders) [121]. There was no significant difference in adverse events and severe adverse events compared with placebo or ustekinumab, however mucocutaneus candida infections were more often seen in patients receiving brodalumab compared with ustekinumab and placebo [121,122].

In Japanese patients a phase II randomized, placebo-controlled study have been conducted with a PASI75 response of 94.6% at week 12 [123].

In AMAGINE 1 and 2, two patients in each study committed suicide while on brodalumab [121,122]. Because events of suicidal ideation and behavior were registered in the clinical trials with brodalumab, additional monitoring for suicidal ideation and depression were implemented as wells as a retrospective data review. A total of six patients committed suicide during the clinical trial program (including trials for PsA and rheumatoid arthritis). A FDA advisory committee was set up to bring light upon the concern about a causative relation between brodalumab and suicide. The committee emphasized the fact that among patients with psoriasis, baseline prevalence of depression was increased, and compared with the general population individuals with psoriasis have higher hazard ratios for depression, anxiety, and suicidality. It was also mentioned, that in the clinical studies, patients with histories of drug and alcohol abuse, depression, and suicidality were not specifically excluded from the clinical trials. This differentiated the brodalumab program from most other biologic studies in psoriasis. No biological mechanism has been described for an association between brodalumab and development of suicidal behavior. 

In February 2017, brodalumab was approved by the FDA. However, because of the observed cases of suicidal ideation and behavior, the label includes a boxed warning. Furthermore, the drug is only available through a restricted program under a Risk Evaluation and Mitigation Strategy called the Siliq REMS Program. This program ensures that both pharmacies, prescribers and patients are informed about the risk of depression and suicidality and the preventive strategy if symptoms occur [124]. However, in 2016 brodalumab had already received approval in Japan, and in May 2017 the European Medical Agency has recommended the approval of brodalumab without remarks to the European Commission [125,126].

Il-17 inhibitors are all highly effective treatments for psoriasis with a rapid onset of action. So far, no head-to-head studies have been conducted to compare the three IL-17 inhibitors on the market. In terms of safety, they are all labeled with an increased risk of infection, most frequently mild infections (e.g., upper respiratory tract infection and candida infections). Manageable mucocutaneous candida infection is associated with all the IL-17 inhibitors, which is probably due to the key role of IL-17 in the host defense against fungi [127,128]. Like TNF-α inhibitors and ustekinumab, it is recommended to screen patients for and treat latent TB before initiating therapy with an IL-17 inhibitor. There have been no results so far from clinical studies, that indicate an association between IL-17 inhibitors and development of active TB. However, the relationship is difficult to examine from these studies, as patients with active TB were excluded and latent TB was treated before initiating treatment with an IL-17 inhibitor [129].

Neutropenia was observed in patients receiving an IL-17 inhibitor, which is in accordance with previous studies that indicate IL-17 to play a role in the regulation of peripheral neutrophils [130]. In patients treated with secukinumab for a 52-week period, 77% of newly developed or worsening neutropenia was grade 1 [104]. In patients treated with ixekizumab week 0–60, 8.6% developed grade 1 neutropenia [108]. In addition, in patients treated with brodalumab week 0–52, 0.4 per 100 patient-years developed grade 1 or 2 neutropenia [122]. For all the three IL-17 inhibitors, grade 3 and 4 neutropenia were rare (0–0.5%). Neutropenia was often seen to resolve and was generally not associated with concurrent infection.

A warning about an association between the IL-17 inhibitors and exacerbation of existing Crohn’s disease has also been included in the labels.

Brodalumab is labeled with relatively more frequent, but mild adverse events compared with secukinumab and ixekizumab, including arthralgia, headache, fatigue, and oropharyngeal pain [131].

One phase III study examining effect and safety of brodalumab in patients with PsA was terminated due to sponsors decision (www.clinicaltrial.gov accessed June 2017: NCT02029495). Another phase III study has been completed, but results have not yet been published (www.cinicaltrial.gov accessed June 2017: NCT02024646).

Only limited long-term safety data is available for the IL-17 inhibitors and so post-marketing surveillance should be performed for any adverse events associated with the use of these new biologics.

### 4.5. Upcoming Biologics

Tildrakizumab and risankizumab are monoclonal antibodies in phase III clinical trials, that target the subunit p19 of IL-23. By doing so, the antibodies exclusively target IL-23 without interfering with the subunit p40 that is shared with IL-12. This is in contrast to ustekinumab which targets both IL-12 and IL-23. The rationale for sparing the IL-12/Th1 pathway is supported by data demonstrating divergent effects of IL-12 and IL-23, with IL-12 signaling resulting in anti-psoriatic effects in the Imiquimod induced psoriasis-like skin inflammation model [132]. Moreover, a better adverse event profile of IL-23p19 inhibitors may be expected by leaving the Th1 response intact for bacterial and viral defense. In a phase II study comparing risankizumab with ustekinumab, results on efficacy is in favor of risankizumab, both at the level of PASI90 and PASI100 [133]. These results support the idea that the IL-23/Th17 axis is the dominant pathway in psoriasis pathogenesis.

Guselkumab, another IL-23p19 neutralizing antibody, was approved by the FDA in July 2017 and results from the two first phase III studies have been published, both controlled for placebo as well as for adalimumab. The largest absolute differences were seen at the level of PASI90: Guselkumab 100 mg week 0 and 4 and then every 8 weeks, resulted in a PASI90 response at week 16 of 73.3% and 70.0% (guselkumab) versus 49.7% and 46.8% (adalimumab) versus 2.9% and 2.4% (placebo) [134,135]. A third phase III study explores switching to guselkumab in patients with non-adequate responses to ustekinumab. Results have not yet been published (www.cinicaltrial.gov accessed June 2017: NCT02203032).

Phase III results for tildrakizumab and risankizumab have not yet been published (as of June 2017). Certolizumab Pegol, a PEGylated TNF-α inhibitor, has for several years been approved for PsA, and has now entered phase III trials for psoriasis. Multiple biologics are in early phase clinical trials for treatment of moderate to severe psoriasis (Table 2).

## 5. Biosimilars

Biosimilars are biologics that are similar but not identical to the original biologic. This is in contrast to generic drugs that consist of identical active compounds of its reference drug and only varies in the vehicle. This difference, is due to the fact, that production of biologics involves living organisms yielding highly complex molecules while generic drugs are produced by a chemical manufacturing process [136]. As the oldest original biologics for the treatment of psoriasis went off patent, biosimilars have emerged. For the TNF-α inhibitors, adalimumab, etanercept, and infliximab, biosimilar therapies have been approved by the FDA. As of May 2017, more biosimilars are in phase III studies to evaluate similarity compared with its originator in patients with psoriasis: CHS-2014 (etanercept biosimilar; www.cinicaltrial.gov: NCT02134210) and BCD-057 and M923 (adalimumab biosimilar; www.cinicaltrial.gov: NCT02762955 and NCT02581345).

Concern has been expressed that biosimilars may not perform with equivalent efficacy and safety as the originators upon their approval and initiation of clinical use. Clinical trials for biosimilars have been conducted with smaller sample size than required for approval of novel biologics. Moreover, clinical trials for each indication of the originator are not necessary for approval of the biosimilar for the same indications. This indication extrapolation bears the risk that a biosimilar may perform with different efficacy and safety compared with the originator in the diseases where the biosimilar has not been tested [137]. Another concern to be addressed, is the clinical situation of switching a patient on the original product to a biosimilar, rather than staying on the original product or initiating biological therapy with a biosimilar. A recently published phase IV trail, the NOR-SWITCH trial, has been conducted for switching of infliximab to the biosimilar CT-P13 among patients with different inflammatory diseases. It was concluded that CT-P13 was not inferior to infliximab at a non-inferiority margin of 15%. However, the study was not powered to conduct analysis on individual diseases [138].

The advantages gained from less data needed for approval, include lower cost of production and thereby less expensive therapies that may increase drug availability for more patients. Along with more biologics losing patent rights, more biosimilars are expected in the future. Under these terms, post-marketing surveillance is of utmost importance for the identification of differences in efficacy and safety that may not have been revealed upon clinical development.

## 6. Conclusions

The major progress made within treatment of moderate to severe psoriasis has been accomplished with the introduction of biologics. Especially, the most recently approved IL-17- and IL-23-targeting biologics proved highly efficient and have a good safety profile in short-term clinical trials. However, only long-term results from clinical studies and post-marketing surveillance can decide to what extent the new therapies are a success. With the development of biosimilars, a question to be addressed is also to which indications systemic therapy should or should not be extended. Aggressive, targeted therapy of mild psoriasis at an early stage also remains unexplored.

Guidance on which of the biologics to choose for the highest rate of success for the individual patient, and how to proceed or discontinue treatment in patients that have achieved effect on biologics seems also warranted. Future studies on the mechanistic link of genetics and co-morbidities to psoriasis may bring information that permits a more individualized treatment approach.

## Figures and Tables

**Table 1:** Biologics approved for psoriasis by the United States Food and Drug Administration as of June 2017.

Biologic Drug	Target	Administration	Treatment Algorithm	Stage of Development	Approved for Psoriasis Arthritis	Withdrawn
Alefacept	LFA ^1^-3	Intra-muscular	15 mg once weekly for 12 weeks	Approved 2003		2011
Efalizumab	CD ^2^11a	Subcutaneous	0.7 mg/kg initial dose, then 1 mg/kg (max 200 mg) once weekly	Approved 2003		2009
Etanercept	TNF ^3^-α	Subcutaneous	50 mg twice weekly for 12 weeks, then 50 mg once weekly	Approved 2004	+	
Infliximab	TNF-α	Intra-venous	5 mg/kg on week 0, 2 and 6, then every 8 weeks	Approved 2006	+	
Adalimumab	TNF-α	Subcutaneous	80 mg initial dose, then 40 mg every 2 weeks, starting one week after initial dose	Approved 2008	+	
Ustekinumab	IL-12/IL-23 p40	Subcutaneous	45 mg (≤100 kg) or 90 mg (>100 kg) on week 0 and 4, then every 12 weeks	Approved 2009	+	
Secukinumab	IL-17A	Subcutaneous	300 mg on week 0, 1, 2, 3, and 4 followed by 300 mg every 4 weeks	Approved 2015	+	
Ixekizumab	IL-17A	Subcutaneous	160 mg week 0, then 80 mg week 2, 4, 6, 8, 10, 12, then 80 mg every 4 weeks	Approved 2016		
Brodalumab	IL-17A receptor	Subcutaneous	210 mg on week 0, 1, and 2, then every 2 weeks	Approved 2017		

^1^ LFA: Lymphocyte function-associated antigen. ^2^ CD: Cluster of differentiation. ^3^ TNF: Tumor necrosis factor.

**Table 2:** Biologics under development for psoriasis as of June 2017. Search at www.clinicaltrial.gov.

Biologic	Type	Target	Stage of Development	www.clinicaltrial.gov
Guselkumab	Human Ig ^1^G1 monoclonal antibody	IL ^2^-23p19	Approved	
Tildrakizumab	Humanized Ig ^1^G1 monoclonal antibody	IL-23p19	Phase III	NCT01722331 NCT01729754
Risankizumab	Humanized IgG1 monoclonal antibody	IL-23p19	Phase III	NCT03047395 NCT02672852 NCT02684370 NCT02694523 NCT02684357
Certolizumab Pegol	PEGylated Fab’ fragment of a humanized IgG1 monoclonal antibody	TNF-α	Phase III	NCT02326298 NCT02326272 NCT02346240
Bimekizumab	Humanized IgG1 monoclonal antibody	IL-17A and IL-17F	Phase II	NCT03025542 NCT03010527 NCT02905006
Neihulizumab	Humanized monoclonal antibody	CD ^3^162 on T-cells	Phase II	NCT02223039 NCT01855880
CJM112	Human monoclonal antibody	IL-17A	Phase II	NCT01828086
Namilumab	Human IgG1 monoclonal antibody	GM-CSF ^4^	Phase II	NCT02129777
Mirikizumab	Humanized monoclonal antibody	IL-23p19	Phase II	NCT02899988
TAB08	Humanized IgG4 monoclonal antibody	CD28 on T-cells	Phase II	NCT02796053
GSK2831781	Humanized antibody dependent cell cytotoxicity enhanced monoclonal afucosylated IgG1antibody	Lymphocyte activation gene-3	Phase I	NCT02195349
T1h	Humanized IgG1 monoclonal antibody	CD6	Phase I	NCT02649270
MSB0010841	Nanobody	IL-17A and IL-17F	Phase I	NCT02156466

^1^ Ig: Immunoglobulin. ^2^ IL: Interleukin. ^3^ CD: Cluster of Differentiation. ^4^ GM-CSF: Granulocyte-macrophage colony-stimulating factor.

## Abbreviations

PsA	Psoriasis arthritis
PASI	Psoriasis area severity index
TNF	Tumor necrosis factor
IL	Interleukin
DNA	Deoxyribonucleic acid
IFN	Interferon
CD	Cluster of differentiation
CXCL	Chemokine (C–X–C motif) ligand
CCL	Chemokine (C–C motif) ligand
FDA	Food and Drug Administration
PGA	Physician Global Assessment
LFA	Lymphocyte function–associated antigen
Ig	Immunoglobulin
APC	Antigen presenting cell
I.M.	Intra-muscular
I.V.	Intra-venous
ICAM	Intercellular Adhesion Molecule
PCL	Progressive multifocal leukoencephalopathy
ADA	Anti-drug-antibodies
TB	Tuberculosis

## References

[1] Bo K., Thoresen M., Dalgard F. (2008). Smokers report more psoriasis, but not atopic dermatitis or hand eczema: Results from a norwegian population survey among adults. Dermatology.

[2] Tsai T.F., Wang T.S., Hung S.T., Tsai P.I., Schenkel B., Zhang M., Tang C.H. (2011). Epidemiology and comorbidities of psoriasis patients in a national database in taiwan. J. Dermatol. Sci..

[3] Madland T.M., Apalset E.M., Johannessen A.E., Rossebo B., Brun J.G. (2005). Prevalence, disease manifestations, and treatment of psoriatic arthritis in western norway. J. Rheumatol..

[4] Zachariae H., Zachariae R., Blomqvist K., Davidsson S., Molin L., Mork C., Sigurgeirsson B. (2002). Quality of life and prevalence of arthritis reported by 5795 members of the nordic psoriasis associations. Data from the nordic quality of life study. Acta Derm. Venereol..

[5] Persson P.G., Leijonmarck C.E., Bernell O., Hellers G., Ahlbom A. (1993). Risk indicators for inflammatory bowel disease. Int. J. Epidemiol..

[6] Boffetta P., Gridley G., Lindelof B. (2001). Cancer risk in a population-based cohort of patients hospitalized for psoriasis in sweden. J. Investig. Dermatol..

[7] Hjuler K.F., Bottcher M., Vestergaard C., Deleuran M., Raaby L., Botker H.E., Iversen L., Kragballe K. (2015). Increased prevalence of coronary artery disease in severe psoriasis and severe atopic dermatitis. Am. J. Med..

[8] Hjuler K.F., Gormsen L.C., Vendelbo M.H., Egeberg A., Nielsen J., Iversen L. (2017). Increased global arterial and subcutaneous adipose tissue inflammation in patients with moderate-to-severe psoriasis. Br. J. Dermatol..

[9] Mehta N.N., Azfar R.S., Shin D.B., Neimanns A.L., Troxel A.B., Gelfand J.M. (2010). Patients with severe psoriasis are at increased risk of cardiovascular mortality: Cohort study using the general practice research database. Eur. Heart J..

[10] Neimann A.L., Shin D.B., Wang X., Margolis D.J., Troxel A.B., Gelfand J.M. (2006). Prevalence of cardiovascular risk factors in patients with psoriasis. J. Am. Acad. Dermatol..

[11] Gelfand J.M., Neimann A.L., Shin D.B., Wang X., Margolis D.J., Troxel A.B. (2006). Risk of myocardial infarction in patients with psoriasis. JAMA.

[12] Abuabara K., Azfar R.S., Shin D.B., Neimann A.L., Troxel A.B., Gelfand J.M. (2010). Cause-specific mortality in patients with severe psoriasis: A population-based cohort study in the uk. Br. J. Dermatol..

[13] Hansson G.K. (2005). Inflammation, atherosclerosis, and coronary artery disease. N. Engl. J. Med..

[14] Saurat J.H., Stingl G., Dubertret L., Papp K., Langley R.G., Ortonne J.P., Unnebrink K., Kaul M., Camez A., Investigators C.S. (2008). Efficacy and safety results from the randomized controlled comparative study of adalimumab vs. Methotrexate vs. Placebo in patients with psoriasis (champion). Br. J. Dermatol..

[15] Warren R.B., Mrowietz U., von Kiedrowski R., Niesmann J., Wilsmann-Theis D., Ghoreschi K., Zschocke I., Falk T.M., Blodorn-Schlicht N., Reich K. (2017). An intensified dosing schedule of subcutaneous methotrexate in patients with moderate to severe plaque-type psoriasis (metop): A 52 week, multicentre, randomised, double-blind, placebo-controlled, phase 3 trial. Lancet.

[16] Dauden E., Puig L., Ferrandiz C., Sanchez-Carazo J.L., Hernanz-Hermosa J.M., Spanish Psoriasis Group of the Spanish Academy of Dermatology and Venereology (2016). Consensus document on the evaluation and treatment of moderate-to-severe psoriasis: Psoriasis group of the spanish academy of dermatology and venereology. J. Eur. Acad. Dermatol. Venereol..

[17] Mueller W., Herrmann B. (1979). Cyclosporin a for psoriasis. N. Engl. J. Med..

[18] Wrone-Smith T., Nickoloff B.J. (1996). Dermal injection of immunocytes induces psoriasis. J. Clin. Investig..

[19] U.S. Food and Drug Administration What Are “Biologics” Questions and Answers. http://www.webcitation.org/6rGBDZ9br.

[20] Torres T., Filipe P. (2015). Small molecules in the treatment of psoriasis. Drug Dev. Res..

[21] Morrow T., Felcone L.H. (2004). Defining the difference: What makes biologics unique. Biotechnol. Healthc..

[22] World Health Organization International Nonproprietary Names (inn) for Biological and Biotechnological Substances. http://www.webcitation.org/6rGAZvnd2.

[23] Di Cesare A., Di Meglio P., Nestle F.O. (2009). The il-23/th17 axis in the immunopathogenesis of psoriasis. J. Investig. Dermatol..

[24] Harrington L.E., Hatton R.D., Mangan P.R., Turner H., Murphy T.L., Murphy K.M., Weaver C.T. (2005). Interleukin 17-producing CD4+ effector T cells develop via a lineage distinct from the T helper type 1 and 2 lineages. Nat. Immunol..

[25] Cai Y., Shen X., Ding C., Qi C., Li K., Li X., Jala V.R., Zhang H.G., Wang T., Zheng J. (2011). Pivotal role of dermal il-17-producing gammadelta t cells in skin inflammation. Immunity.

[26] Mahil S.K., Capon F., Barker J.N. (2016). Update on psoriasis immunopathogenesis and targeted immunotherapy. Semin. Immunopathol..

[27] Ortega C., Fernandez S., Carrillo J.M., Romero P., Molina I.J., Moreno J.C., Santamaria M. (2009). Il-17-producing CD8(+) t lymphocytes from psoriasis skin plaques are cytotoxic effector cells that secrete th17-related cytokines. J. Leukoc. Biol..

[28] Johnston A., Fritz Y., Dawes S.M., Diaconu D., Al-Attar P.M., Guzman A.M., Chen C.S., Fu W., Gudjonsson J.E., McCormick T.S. (2013). Keratinocyte overexpression of il-17c promotes psoriasiform skin inflammation. J. Immunol..

[29] Nestle F.O., Kaplan D.H., Barker J. (2009). Psoriasis. N. Engl. J. Med..

[30] Griffiths C.E., Reich K., Lebwohl M., van de Kerkhof P., Paul C., Menter A., Cameron G.S., Erickson J., Zhang L., Secrest R.J. (2015). Comparison of ixekizumab with etanercept or placebo in moderate-to-severe psoriasis (uncover-2 and uncover-3): Results from two phase 3 randomised trials. Lancet.

[31] Puig L. (2015). Pasi90 response: The new standard in therapeutic efficacy for psoriasis. J. Eur. Acad. Dermatol. Venereol..

[32] Brimhall A.K., King L.N., Licciardone J.C., Jacobe H., Menter A. (2008). Safety and efficacy of alefacept, efalizumab, etanercept and infliximab in treating moderate to severe plaque psoriasis: A meta-analysis of randomized controlled trials. Br. J. Dermatol..

[33] Jenneck C., Novak N. (2007). The safety and efficacy of alefacept in the treatment of chronic plaque psoriasis. Ther. Clin. Risk Manag..

[34] Krueger G.G. (2004). The remittive effects of alefacept. J. Cutan. Med. Surg..

[35] Krueger G.G., Papp K.A., Stough D.B., Loven K.H., Gulliver W.P., Ellis C.N., Alefacept Clinical Study Group (2002). A randomized, double-blind, placebo-controlled phase iii study evaluating efficacy and tolerability of 2 courses of alefacept in patients with chronic plaque psoriasis. J. Am. Acad. Dermatol..

[36] Lebwohl M., Christophers E., Langley R., Ortonne J.P., Roberts J., Griffiths C.E., Alefacept Clinical Study Group (2003). An international, randomized, double-blind, placebo-controlled phase 3 trial of intramuscular alefacept in patients with chronic plaque psoriasis. Arch. Dermatol..

[37] Al-Suwaidan S.N., Feldman S.R. (2000). Clearance is not a realistic expectation of psoriasis treatment. J. Am. Acad. Dermatol..

[38] Ahn C.S., Gustafson C.J., Sandoval L.F., Davis S.A., Feldman S.R. (2013). Cost effectiveness of biologic therapies for plaque psoriasis. Am. J. Clin. Dermatol..

[39] Jullien D., Prinz J.C., Langley R.G.B., Caro I., Dummer W., Joshi A., Dedrick R., Natta P. (2004). T-cell modulation for the treatment of chronic plaque psoriasis with efalizumab (raptiva (tm)): Mechanisms of action. Dermatology.

[40] Schon M.P. (2008). Efalizumab in the treatment of psoriasis: Mode of action, clinical indications, efficacy, and safety. Clin. Dermatol..

[41] Dubertret L., Sterry W., Bos J.D., Chimenti S., Shumack S., Larsen C.G., Shear N.H., Papp K.A., CLEAR Multinational Study Group (2006). Clinical experience acquired with the efalizumab (raptiva) (clear) trial in patients with moderate-to-severe plaque psoriasis: Results from a phase iii international randomized, placebo-controlled trial. Br. J. Dermatol..

[42] Menter A., Gordon K., Carey W., Hamilton T., Glazer S., Caro I., Li N., Gulliver W. (2005). Efficacy and safety observed during 24 weeks of efalizumab therapy in patients with moderate to severe plaque psoriasis. Arch. Dermatol..

[43] Leonardi C., Menter A., Hamilton T., Caro I., Xing B., Gottlieb A.B. (2008). Efalizumab: Results of a 3-year continuous dosing study for the long-term control of psoriasis. Br. J. Dermatol..

[44] Seminara N.M., Gelfand J.M. (2010). Assessing long-term drug safety: Lessons (re) learned from raptiva. Semin. Cutan. Med. Surg..

[45] U.S. Food and Drug Administration Fda Statement on the Voluntary Withdrawal of Raptiva from the U.S. Market. http://www.webcitation.org/6rGBTfcbt.

[46] Mease P.J., Goffe B.S., Metz J., VanderStoep A., Finck B., Burge D.J. (2000). Etanercept in the treatment of psoriatic arthritis and psoriasis: A randomised trial. Lancet.

[47] Mease P.J., Kivitz A.J., Burch F.X., Siegel E.L., Cohen S.B., Ory P., Salonen D., Rubenstein J., Sharp J.T., Tsuji W. (2004). Etanercept treatment of psoriatic arthritis: Safety, efficacy, and effect on disease progression. Arthritis Rheumatol..

[48] Prnewswire Fda Approves Expanded Use of Enbrel^®^ (Etanercept) to Treat Children with Chronic Moderate-to-Severe Plaque Psoriasis. http://www.webcitation.org/6rGBpHvS0.

[49] Tyring S., Gottlieb A., Papp K., Gordon K., Leonardi C., Wang A., Lalla D., Woolley M., Jahreis A., Zitnik R. (2006). Etanercept and clinical outcomes, fatigue, and depression in psoriasis: Double-blind placebo-controlled randomised phase iii trial. Lancet.

[50] Leonardi C.L., Powers J.L., Matheson R.T., Goffe B.S., Zitnik R., Wang A., Gottlieb A.B., Etanercept Psoriasis Study Group (2003). Etanercept as monotherapy in patients with psoriasis. N. Engl. J. Med..

[51] Papp K.A., Tyring S., Lahfa M., Prinz J., Griffiths C.E.M., Nakanishi A.M., Zitnik R., van de Kerkhof P.C.M., Grp E.P.S. (2005). A global phase iii randomized controlled trial of etanercept in psoriasis: Safety, efficacy, and effect of dose reduction. Br. J. Dermatol..

[52] Tyring S., Gordon K.B., Poulin Y., Langley R.G., Gottlieb A.B., Dunn M., Jahreis A. (2007). Long-term safety and efficacy of 50 mg of etanercept twice weekly in patients with psoriasis. Arch. Dermatol..

[53] Bachelez H., van de Kerkhof P.C., Strohal R., Kubanov A., Valenzuela F., Lee J.H., Yakusevich V., Chimenti S., Papacharalambous J., Proulx J. (2015). Tofacitinib versus etanercept or placebo in moderate-to-severe chronic plaque psoriasis: A phase 3 randomised non-inferiority trial. Lancet.

[54] Gall J.S., Kalb R.E. (2008). Infliximab for the treatment of plaque psoriasis. Biologics.

[55] Lucka T.C., Pathirana D., Sammain A., Bachmann F., Rosumeck S., Erdmann R., Schmitt J., Orawa H., Rzany B., Nast A. (2012). Efficacy of systemic therapies for moderate-to-severe psoriasis: A systematic review and meta-analysis of long-term treatment. J. Eur. Acad. Dermatol. Venereol..

[56] Menter A., Feldman S.R., Weinstein G.D., Papp K., Evans R., Guzzo C., Li S., Dooley L.T., Arnold C., Gottlieb A.B. (2007). A randomized comparison of continuous vs. Intermittent infliximab maintenance regimens over 1 year in the treatment of moderate-to-severe plaque psoriasis. J. Am. Acad. Dermatol..

[57] Reich K., Nestle F.O., Papp K., Ortonne J.P., Evans R., Guzzo C., Li S., Dooley L.T., Griffiths C.E.M., Investigators E.S. (2005). Infliximab induction and maintenance therapy for moderate-to-severe psoriasis: A phase iii, multicentre, double-blind trial. Lancet.

[58] Yang H.Z., Wang K., Jin H.Z., Gao T.W., Xiao S.X., Xu J.H., Wang B.X., Zhang F.R., Li C.Y., Liu X.M. (2012). Infliximab monotherapy for chinese patients with moderate to severe plaque psoriasis: A randomized, double-blind, placebo-controlled multicenter trial. Chin. Med. J. (Engl.).

[59] Torii H., Nakagawa H., Japanese Infliximab Study Ivestigators (2010). Infliximab monotherapy in japanese patients with moderate-to-severe plaque psoriasis and psoriatic arthritis. A randomized, double-blind, placebo-controlled multicenter trial. J. Dermatol. Sci..

[60] Spertino J., Lopez-Ferrer A., Vilarrasa E., Puig L. (2014). Long-term study of infliximab for psoriasis in daily practice: Drug survival depends on combined treatment, obesity and infusion reactions. J. Eur. Acad. Dermatol. Venereol..

[61] Gottlieb A.B., Evans R., Li S., Dooley L.T., Guzzo C.A., Baker D., Bala M., Marano C.W., Menter A. (2004). Infliximab induction therapy for patients with severe plaque-type psoriasis: A randomized, double-blind, placebo-controlled trial. J. Am. Acad. Dermatol..

[62] Lichtenstein L., Ron Y., Kivity S., Ben-Horin S., Israeli E., Fraser G.M., Dotan I., Chowers Y., Confino-Cohen R., Weiss B. (2015). Infliximab-related infusion reactions: Systematic review. J. Crohn’s Colitis.

[63] Wang Z., Wang J., Fu L., Dong S., Ge Y., Zhang J., Huang B., Wang Q., Wang Z. (2015). Effectiveness and risk associated with infliximab alone and in combination with immunosuppressors for crohn’s disease: A systematic review and meta-analysis. Int. J. Clin. Exp. Med..

[64] Mease P.J., Gladman D.D., Ritchlin C.T., Ruderman E.M., Steinfeld S.D., Choy E.H., Sharp J.T., Ory P.A., Perdok R.J., Weinberg M.A. (2005). Adalimumab for the treatment of patients with moderately to severely active psoriatic arthritis: Results of a double-blind, randomized, placebo-controlled trial. Arthritis Rheumatol..

[65] Menter A., Tyring S.K., Gordon K., Kimball A.B., Leonardi C.L., Langley R.G., Strober B.E., Kaul M., Gu Y., Okun M. (2008). Adalimumab therapy for moderate to severe psoriasis: A randomized, controlled phase iii trial. J. Am. Acad. Dermatol..

[66] Gordon K., Papp K., Poulin Y., Gu Y.H., Rozzo S., Sasso E.H. (2012). Long-term efficacy and safety of adalimumab in patients with moderate to severe psoriasis treated continuously over 3 years: Results from an open-label extension study for patients from reveal. J. Am. Acad. Dermatol..

[67] Asahina A., Nakagawa H., Etoh T., Ohtsuki M., Grp A.M.-S. (2010). Adalimumab in japanese patients with moderate to severe chronic plaque psoriasis: Efficacy and safety results from a phase ii/iii randomized controlled study. J. Dermatol..

[68] Cai L., Gu J., Zheng J., Zheng M., Wang G., Xi L.Y., Hao F., Liu X.M., Sun Q.N., Wang Y. (2017). Efficacy and safety of adalimumab in chinese patients with moderate-to-severe plaque psoriasis: Results from a phase 3, randomized, placebo-controlled, double-blind study. J. Eur. Acad. Dermatol. Venereol..

[69] Nast A., Gisondi P., Ormerod A.D., Saiag P., Smith C., Spuls P.I., Arenberger P., Bachelez H., Barker J., Dauden E. (2015). European S3-guidelines on the systemic treatment of psoriasis vulgaris—Update 2015—Short version—EDF in cooperation with EADV and IPC. J. Eur. Acad. Dermatol. Venereol..

[70] Raaby L., Zachariae C., Ostensen M., Heickendorff L., Thielsen P., Gronbaek H., Skov L., Kyvsgaard N., Madsen J.T., Heidenheim M. (2017). Methotrexate use and monitoring in patients with psoriasis: A consensus report based on a danish expert meeting. Acta Derm. Venereol..

[71] Thaci D., Ortonne J.P., Chimenti S., Ghislain P.D., Arenberger P., Kragballe K., Saurat J.H., Khemis A., Sprogel P., Esslinger H.U. (2010). A phase iiib, multicentre, randomized, double-blind, vehicle-controlled study of the efficacy and safety of adalimumab with and without calcipotriol/betamethasone topical treatment in patients with moderate to severe psoriasis: The believe study. Br. J. Dermatol..

[72] De Vries A.C., Thio H.B., de Kort W.J., Opmeer B.C., van der Stok H.M., de Jong E.M., Horvath B., Busschbach J.J., Nijsten T.E., Spuls P.I. (2017). A prospective randomized controlled trial comparing infliximab and etanercept in patients with moderate-to-severe chronic plaque-type psoriasis: The psoriasis infliximab vs. Etanercept comparison evaluation (piece) study. Br. J. Dermatol..

[73] Schmitt J., Zhang Z., Wozel G., Meurer M., Kirch W. (2008). Efficacy and tolerability of biologic and nonbiologic systemic treatments for moderate-to-severe psoriasis: Meta-analysis of randomized controlled trials. Br. J. Dermatol..

[74] Hsu L., Armstrong A.W. (2013). Anti-drug antibodies in psoriasis: A critical evaluation of clinical significance and impact on treatment response. Expert. Rev. Clin. Immunol..

[75] Kui R., Gal B., Gaal M., Kiss M., Kemeny L., Gyulai R. (2016). Presence of antidrug antibodies correlates inversely with the plasma tumor necrosis factor (tnf)-alpha level and the efficacy of tnf-inhibitor therapy in psoriasis. J. Dermatol..

[76] Prescribing Information, Enbrel (Etanercept). http://www.webcitation.org/6rGCfL3tR.

[77] Prescribing Information, Remicade (Infliximab). http://www.webcitation.org/6rGDV99FL.

[78] Prescribing Information, Humira (Adalimumab). http://www.webcitation.org/6rGCyzYce.

[79] Harris J., Keane J. (2010). How tumour necrosis factor blockers interfere with tuberculosis immunity. Clin. Exp. Immunol..

[80] Ergun T., Seckin D., Baskan Bulbul E., Onsun N., Ozgen Z., Unalan P., Alpsoy E., Karakurt S. (2015). The risk of tuberculosis in patients with psoriasis treated with anti-tumor necrosis factor agents. Int. J. Dermatol..

[81] Report from The Danish Council for the Use of Expensive Hospital Medicines. http://www.regioner.dk/media/1843/bgn-pso-123452401591.pdf.

[82] Leonardi C.L., Kimball A.B., Papp K.A., Yeilding N., Guzzo C., Wang Y.H., Li S., Dooley L.T., Gordon K.B., Investigators P.S. (2008). Efficacy and safety of ustekinumab, a human interleukin-12/23 monoclonal antibody, in patients with psoriasis: 76-week results from a randomised, double-blind, placebo-controlled trial (phoenix 1). Lancet.

[83] Papp K.A., Langley R.G., Lebwohl M., Krueger G.G., Szapary P., Yeilding N., Guzzo C., Hsu M.C., Wang Y.H., Li S. (2008). Efficacy and safety of ustekinumab, a human interleukin-12/23 monoclonal antibody, in patients with psoriasis: 52-week results from a randomised, double-blind, placebo-controlled trial (phoenix 2). Lancet.

[84] Igarashi A., Kato T., Kato M., Song M., Nakagawa H., Grp J.U.S. (2012). Efficacy and safety of ustekinumab in japanese patients with moderate-to-severe plaque-type psoriasis: Long-term results from a phase 2/3 clinical trial. J. Dermatol..

[85] Tsai T.F., Ho J.C., Song M., Szapary P., Guzzo C., Shen Y.K., Li S., Kim K.J., Kim T.Y., Choi J.H. (2011). Efficacy and safety of ustekinumab for the treatment of moderate-to-severe psoriasis: A phase iii, randomized, placebo-controlled trial in taiwanese and korean patients (pearl). J. Dermatol. Sci..

[86] Zhu X., Zheng M., Song M., Shen Y.K., Chan D., Szapary P.O., Wang B., Investigators L. (2013). Efficacy and safety of ustekinumab in chinese patients with moderate to severe plaque-type psoriasis: Results from a phase 3 clinical trial (lotus). J. Drugs Dermatol..

[87] Gniadecki R., Bang B., Bryld L.E., Iversen L., Lasthein S., Skov L. (2015). Comparison of long-term drug survival and safety of biologic agents in patients with psoriasis vulgaris. Br. J. Dermatol..

[88] Van den Reek J.M., Zweegers J., Kievit W., Otero M.E., van Lumig P.P., Driessen R.J., Ossenkoppele P.M., Njoo M.D., Mommers J.M., Koetsier M.I. (2014). ‘Happy’ drug survival of adalimumab, etanercept and ustekinumab in psoriasis in daily practice care: Results from the biocapture network. Br. J. Dermatol..

[89] Warren R.B., Smith C.H., Yiu Z.Z.N., Ashcroft D.M., Barker J.N.W.N., Burden A.D., Lunt M., McElhone K., Ormerod A.D., Owen C.M. (2015). Differential drug survival of biologic therapies for the treatment of psoriasis: A prospective observational cohort study from the british association of dermatologists biologic interventions register (badbir). J. Investig. Dermatol..

[90] Clemmensen A., Spon M., Skov L., Zachariae C., Gniadecki R. (2011). Responses to ustekinumab in the anti-tnf agent-naive vs. Anti-tnf agent-exposed patients with psoriasis vulgaris. J. Eur. Acad. Dermatol..

[91] Kimball A.B., Papp K.A., Wasfi Y., Chan D., Bissonnette R., Sofen H., Yeilding N., Li S., Szapary P., Gordon K.B. (2013). Long-term efficacy of ustekinumab in patients with moderate-to-severe psoriasis treated for up to 5 years in the phoenix 1 study. J. Eur. Acad. Dermatol. Venereol..

[92] Langley R.G., Lebwohl M., Krueger G.G., Szapary P.O., Wasfi Y., Chan D., Hsu M.C., You Y., Poulin Y., Korman N. (2015). Long-term efficacy and safety of ustekinumab, with and without dosing adjustment, in patients with moderate-to-severe psoriasis: Results from the phoenix 2 study through 5 years of follow-up. Br. J. Dermatol..

[93] Griffiths C.E., Strober B.E., van de Kerkhof P., Ho V., Fidelus-Gort R., Yeilding N., Guzzo C., Xia Y., Zhou B., Li S. (2010). Comparison of ustekinumab and etanercept for moderate-to-severe psoriasis. N. Engl. J. Med..

[94] Gordon K.B., Papp K.A., Langley R.G., Ho V., Kimball A.B., Guzzo C., Yeilding N., Szapary P.O., Fakharzadeh S., Li S. (2012). Long-term safety experience of ustekinumab in patients with moderate to severe psoriasis (part ii of ii): Results from analyses of infections and malignancy from pooled phase ii and iii clinical trials. J. Am. Acad. Dermatol..

[95] Prescribing Information, Stelara (Ustekinumab). http://www.webcitation.org/6rKr49YYM.

[96] MacLennan C., Fieschi C., Lammas D.A., Picard C., Dorman S.E., Sanal O., MacLennan J.M., Holland S.M., Ottenhoff T.H.M., Casanova J.L. (2004). Interleukin (il)-12 and il-23 are key cytokines for immunity against salmonella in humans. J. Infect. Dis..

[97] Lynch M., Roche L., Horgan M., Ahmad K., Hackett C., Ramsay B. (2017). Peritoneal tuberculosis in the setting of ustekinumab treatment for psoriasis. JAAD Case Rep..

[98] Kirkham B.W., Kavanaugh A., Reich K. (2014). Interleukin-17a: A unique pathway in immune-mediated diseases: Psoriasis, psoriatic arthritis and rheumatoid arthritis. Immunology.

[99] Langley R.G., Elewski B.E., Lebwohl M., Reich K., Griffiths C.E., Papp K., Puig L., Nakagawa H., Spelman L., Sigurgeirsson B. (2014). Secukinumab in plaque psoriasis--results of two phase 3 trials. N. Engl. J. Med..

[100] Blauvelt A., Prinz J.C., Gottlieb A.B., Kingo K., Sofen H., Ruer-Mulard M., Singh V., Pathan R., Papavassilis C., Cooper S. (2015). Secukinumab administration by pre-filled syringe: Efficacy, safety and usability results from a randomized controlled trial in psoriasis (feature). Br. J. Dermatol..

[101] Paul C., Lacour J.P., Tedremets L., Kreutzer K., Jazayeri S., Adams S., Guindon C., You R., Papavassilis C., Grp J.S. (2015). Efficacy, safety and usability of secukinumab administration by autoinjector/pen in psoriasis: A randomized, controlled trial (juncture). J. Eur. Acad. Dermatol..

[102] Blauvelt A., Reich K., Tsai T.F., Tyring S., Vanaclocha F., Kingo K., Ziv M., Pinter A., Vender R., Hugot S. (2017). Secukinumab is superior to ustekinumab in clearing skin of subjects with moderate-to-severe plaque psoriasis up to 1 year: Results from the clear study. J. Am. Acad. Dermatol..

[103] Thaci D., Blauvelt A., Reich K., Tsai T.F., Vanaclocha F., Kingo K., Ziv M., Pinter A., Hugot S., You R.Q. (2015). Secukinumab is superior to ustekinumab in clearing skin of subjects with moderate to severe plaque psoriasis: Clear, a randomized controlled trial. J. Am. Acad. Dermatol..

[104] Van de Kerkhof P.C., Griffiths C.E., Reich K., Leonardi C.L., Blauvelt A., Tsai T.F., Gong Y., Huang J., Papavassilis C., Fox T. (2016). Secukinumab long-term safety experience: A pooled analysis of 10 phase ii and iii clinical studies in patients with moderate to severe plaque psoriasis. J. Am. Acad. Dermatol..

[105] U.S. Food and Drug Administartion Fda Approves New Psoriasis Drug Taltz. http://www.webcitation.org/6rGDjK5VG.

[106] Mease P.J., van der Heijde D., Ritchlin C.T., Okada M., Cuchacovich R.S., Shuler C.L., Lin C.Y., Braun D.K., Lee C.H., Gladman D.D. (2017). Ixekizumab, an interleukin-17a specific monoclonal antibody, for the treatment of biologic-naive patients with active psoriatic arthritis: Results from the 24-week randomised, double-blind, placebo-controlled and active (adalimumab)-controlled period of the phase iii trial spirit-p1. Ann. Rheum. Dis..

[107] Nash P., Kirkham B., Okada M., Rahman P., Combe B., Burmester G.R., Adams D.H., Kerr L., Lee C., Shuler C.L. (2017). Ixekizumab for the treatment of patients with active psoriatic arthritis and an inadequate response to tumour necrosis factor inhibitors: Results from the 24-week randomised, double-blind, placebo-controlled period of the spirit-p2 phase 3 trial. Lancet.

[108] Gordon K.B., Blauvelt A., Papp K.A., Langley R.G., Luger T., Ohtsuki M., Reich K., Amato D., Ball S.G., Braun D.K. (2016). Phase 3 trials of ixekizumab in moderate-to-severe plaque psoriasis. N. Engl. J. Med..

[109] Saeki H., Nakagawa H., Ishii T., Morisaki Y., Aoki T., Berclaz P.Y., Heffernan M. (2015). Efficacy and safety of open-label ixekizumab treatment in japanese patients with moderate-to-severe plaque psoriasis, erythrodermic psoriasis and generalized pustular psoriasis. J. Eur. Acad. Dermatol..

[110] Saeki H., Nakagawa H., Nakajo K., Ishii T., Morisaki Y., Aoki T., Cameron G.S., Osuntokun O.O., Grp J.I.S. (2017). Efficacy and safety of ixekizumab treatment for japanese patients with moderate to severe plaque psoriasis, erythrodermic psoriasis and generalized pustular psoriasis: Results from a 52-week, open-label, phase 3 study (uncover-j). J. Dermatol..

[111] Blauvelt A., Papp K.A., Griffiths C.E., Puig L., Weisman J., Dutronc Y., Kerr L.F., Ilo D., Mallbris L., Augustin M. (2017). Efficacy and safety of switching to ixekizumab in etanercept non-responders: A subanalysis from two phase iii randomized clinical trials in moderate-to-severe plaque psoriasis (uncover-2 and -3). Am. J. Clin. Dermatol..

[112] Menter A., Warren R.B., Langley R.G., Merola J.F., Kerr L.N., Dennehy E.B., Shrom D., Amato D., Okubo Y., Reich K. (2017). Efficacy of ixekizumab compared to etanercept and placebo in patients with moderate-to-severe plaque psoriasis and non-pustular palmoplantar involvement: Results from three phase 3 trials (uncover-1, uncover-2 and uncover-3). J. Eur. Acad. Dermatol. Venereol..

[113] Armstrong A.W., Lynde C.W., McBride S.R., Stahle M., Edson-Heredia E., Zhu B., Amato D., Nikai E., Yang F.E., Gordon K.B. (2016). Effect of ixekizumab treatment on work productivity for patients with moderate-to-severe plaque psoriasis: Analysis of results from 3 randomized phase 3 clinical trials. JAMA Dermatol..

[114] Strober B., Leonardi C., Papp K.A., Mrowietz U., Ohtsuki M., Bissonnette R., Ferris L.K., Paul C., Lebwohl M., Braun D.K. (2017). Short- and long-term safety outcomes with ixekizumab from 7 clinical trials in psoriasis: Etanercept comparisons and integrated data. J. Am. Acad. Dermatol..

[115] Gaffen S.L. (2009). Structure and signalling in the il-17 receptor family (vol 9, pg 556, 2009). Nat. Rev. Immunol..

[116] Martin D.A., Towne J.E., Kricorian G., Klekotka P., Gudjonsson J.E., Krueger J.G., Russell C.B. (2013). The emerging role of il-17 in the pathogenesis of psoriasis: Preclinical and clinical findings. J. Investig. Dermatol..

[117] Fujishima S., Watanabe H., Kawaguchi M., Suzuki T., Matsukura S., Homma T., Howell B.G., Hizawa N., Mitsuya T., Huang S.K. (2010). Involvement of il-17f via the induction of il-6 in psoriasis. Arch. Dermatol. Res..

[118] Watanabe H., Kawaguchi M., Fujishima S., Ogura M., Matsukura S., Takeuchi H., Ohba M., Sueki H., Kokubu F., Hizawa N. (2009). Functional characterization of il-17f as a selective neutrophil attractant in psoriasis. J. Investig. Dermatol..

[119] Bertelsen T., Ljungberg C., Kjellerup R., Iversen L., Johansen C. (2016). Il-17f regulates psoriasis-associated genes through i kappa b zeta. J. Investig. Dermatol..

[120] Johansen C., Usher P.A., Kjellerup R.B., Lundsgaard D., Iversen L., Kragballe K. (2009). Characterization of the interleukin-17 isoforms and receptors in lesional psoriatic skin. Br. J. Dermatol..

[121] Lebwohl M., Strober B., Menter A., Gordon K., Weglowska J., Puig L., Papp K., Spelman L., Toth D., Kerdel F. (2015). Phase 3 studies comparing brodalumab with ustekinumab in psoriasis. N. Engl. J. Med..

[122] Papp K.A., Reich K., Paul C., Blauvelt A., Baran W., Bolduc C., Toth D., Langley R.G., Cather J., Gottlieb A.B. (2016). A prospective phase iii, randomized, double-blind, placebo-controlled study of brodalumab in patients with moderate-to-severe plaque psoriasis. Br. J. Dermatol..

[123] Nakagawa H., Niiro H., Ootaki K., Japanese Brodalumab Study Group (2016). Brodalumab, a human anti-interleukin-17-receptor antibody in the treatment of japanese patients with moderate-to-severe plaque psoriasis: Efficacy and safety results from a phase ii randomized controlled study. J. Dermatol. Sci..

[124] U.S. Food and Drug Administration Fda Approves New Psoriasis Drug. http://www.webcitation.org/6rGE8u2CY.

[125] European Medicines Agency Kyntheum. http://www.webcitation.org/6rGERhJc2.

[126] Kyowa Kirin Lumicef^®^ Approved in Japan. http://www.webcitation.org/6rGEdu4hs.

[127] Conti H.R., Shen F., Nayyar N., Stocum E., Sun J.N., Lindemann M.J., Ho A.W., Hai J.H., Yu J.J., Jung J.W. (2009). Th17 cells and il-17 receptor signaling are essential for mucosal host defense against oral candidiasis. J. Exp. Med..

[128] Puel A., Cypowyj S., Bustamante J., Wright J.F., Liu L.Y., Lim H.K., Migaud M., Israel L., Chrabieh M., Audry M. (2011). Chronic mucocutaneous candidiasis in humans with inborn errors of interleukin-17 immunity. Science.

[129] Tsai T.F., Blauvelt A., Gong Y.K., Huang J.Q., Fox T. (2015). Secukinumab treatment shows no evidence for reactivation of previous or latent tb infection in subjects with psoriasis: A pooled phase 3 safety analysis. J. Am. Acad. Dermatol..

[130] Forlow S.B., Schurr J.R., Kolls J.K., Bagby G.J., Schwarzenberger P.O., Ley K. (2001). Increased granulopoiesis through interleukin-17 and granulocyte colony-stimulating factor in leukocyte adhesion molecule-deficient mice. Blood.

[131] Prescribing Information, Brodalumab. http://www.webcitation.org/6rKuHGiye.

[132] Kulig P., Musiol S., Freiberger S.N., Schreiner B., Gyulveszi G., Russo G., Pantelyushin S., Kishihara K., Alessandrini F., Kundig T. (2016). Il-12 protects from psoriasiform skin inflammation. Nat. Commun..

[133] Papp K.A., Blauvelt A., Bukhalo M., Gooderham M., Krueger J.G., Lacour J.P., Menter A., Philipp S., Sofen H., Tyring S. (2017). Risankizumab versus ustekinumab for moderate-to-severe plaque psoriasis. N. Engl. J. Med..

[134] Blauvelt A., Papp K.A., Griffiths C.E., Randazzo B., Wasfi Y., Shen Y.K., Li S., Kimball A.B. (2017). Efficacy and safety of guselkumab, an anti-interleukin-23 monoclonal antibody, compared with adalimumab for the continuous treatment of patients with moderate to severe psoriasis: Results from the phase iii, double-blinded, placebo- and active comparator-controlled voyage 1 trial. J. Am. Acad. Dermatol..

[135] Reich K., Armstrong A.W., Foley P., Song M., Wasfi Y., Randazzo B., Li S., Shen Y.K., Gordon K.B. (2017). Efficacy and safety of guselkumab, an anti-interleukin-23 monoclonal antibody, compared with adalimumab for the treatment of patients with moderate to severe psoriasis with randomized withdrawal and retreatment: Results from the phase iii, double-blind, placebo- and active comparator-controlled voyage 2 trial. J. Am. Acad. Dermatol..

[136] Weise M., Bielsky M.C., De Smet K., Ehmann F., Ekman N., Giezen T.J., Gravanis I., Heim H.K., Heinonen E., Ho K. (2012). Biosimilars: What clinicians should know. Blood.

[137] Blauvelt A., Puig L., Chimenti S., Vender R., Rajagopalan M., Romiti R., Skov L., Zachariae C., Young H., Prens E. (2016). Biosimilars for psoriasis: Clinical studies to determine similarity. Br. J. Dermatol..

[138] Jorgensen K.K., Olsen I.C., Goll G.L., Lorentzen M., Bolstad N., Haavardsholm E.A., Lundin K.E.A., Mork C., Jahnsen J., Kvien T.K. (2017). Switching from originator infliximab to biosimilar ct-p13 compared with maintained treatment with originator infliximab (nor-switch): A 52-week, randomised, double-blind, non-inferiority trial. Lancet.

